# Questionnaire development on measuring parents’ anxiety about their children’s education: Empirical evidence of parental perceived anxiety data for primary and secondary school students in China

**DOI:** 10.3389/fpsyg.2022.1018313

**Published:** 2022-10-12

**Authors:** Jiangjie Sun, Tong Liu, Yufei Gao, Hui Li, Yao Chen, Haibao Diao, Genfa Zhang, Hui Shen, Rong Chang, Zhenliang Yu, Jingru Lu, Liang Liang, Liping Zhang

**Affiliations:** ^1^School of Management, Hefei University of Technology, Hefei, China; ^2^Health Management College, Anhui Medical University, Hefei, China; ^3^Hefei No. 42 Middle School, Hefei, China; ^4^Hefei No. 1 Middle School, Hefei, China; ^5^Feixi Experimental Senior Secondary School, Hefei, China; ^6^Anhui Hefei No. 6 High School, Hefei, China; ^7^Hefei No. 45 Middle School Furong Branch South District, Hefei, China; ^8^The 4th Primary School Affiliated to Hefei Normal School, Hefei, China; ^9^School of Marxism, Anhui Medical University, Hefei, China

**Keywords:** primary and secondary students, PAE, MQPAE, measure, reliability and validity

## Abstract

**Background:**

With the implementation of the “double reduction” policy in China, parents of primary and secondary school students are experiencing a growing trend of educational anxiety that needs to be alleviated.

**Objective:**

To manage the education anxiety risk of parents of primary and secondary school students, a *measurement questionnaire of parents’ anxiety about their children’s education* (MQPAE) was developed and its reliability and validity were evaluated.

**Methods:**

A self-administered MQPAE was developed. An online crowdsourcing questionnaire platform was used to collect data on *parents’ anxiety about their children’s education* (PAE), and parents of primary and secondary school students in Hefei, China, were selected as the study population. The randomly extracted 5,747 questionnaires were gradually screened by discrete trend method, *t*-test, and Pearson’s correlation coefficient method for the initial screening of PAE items, based on which exploratory factor analysis (EFA) was conducted for the final screening of questionnaire items and the reliability of the questionnaire. The reliability of the questionnaire was assessed by internal consistency and Pearson’s correlation coefficient analysis. Confirmatory factor analysis (CFA) was conducted using 639 pre-selected data to investigate the validity of the questionnaire. Structural equation modeling was used to investigate the structural validity of the questionnaire, and average variance extracted (AVE), combined reliability (CR), and maximum of shared squared variance (MSV) were used to test for convergent and discriminant validity.

**Results:**

Exploratory factor analysis extracted five factors with a cumulative variance contribution of 65.66%. The CFA showed that χ^2^/df = 4.306, CFI = 0.920, NFI = 0.898, RMSEA = 0.072<0.08, AGFI = 0.839>0.80, PNFI = 0.793 and PGFI = 0.708. The overall Cronbach’s α coefficient of the questionnaire was 0.956, and the factors’ Cronbach’s α coefficients were 0.926, 0.857, 0.913, 0.901, and 0.768, respectively. Repeated measurements of Pearson’s correlation coefficients were 0.908, 0.911, 0.873, 0.891, 0.907 and 0.885 (all *p* < 0.001). The AVE was greater than 0.5 and the CR was greater than 0.7, and the value of the MSV was less than the corresponding AVE.

**Conclusion:**

The MQPAE has good reliability and validity and can be used in studies related to PAE of primary and secondary school students.

## Introduction

In the 1970s, driven by the rapid economic development, the demand of education and training for primary and secondary school students led to the profit-seeking of out-of-school training institutions. The issue of educational equity contributed to the pathological development of PAE of primary and secondary school students, leading to the ultimate failure of the “double reduction” policy in Korea (Ji, 2022). History will not repeat itself, but it will press a similar rhyme. At present, China’s booming economic and technological development, and high material consumption drive the surge of real estate and education consumption. The hot pursuit of the tutoring industry and “school districts” brings parents anxiety about the “education race” with the goal of further education (Zhou, 2021), which worsens the problem of “low fertility” of the society and contradicts the current situation of “aging” in China. Studies have confirmed that load reduction has a positive effect on improving learning outcomes and promoting development of education quality (Zhou, 2021). Therefore, “load reduction” is an effective means to promote high-quality development of basic education, which is consistent with the need of the modern era in China.

In China, the most stringent “double reduction” campaign in history began in July 2021, when the State Council issued the “Opinions on Further Reducing the Burden of Homework and Off-Campus Training for Students in Compulsory Education” which was to clarify the direction and responsibility of reducing the educational burden from the institutional perspective. Since the implementation of the “double reduction” policy in Hefei, Anhui Province, a total of 1,142 out-of-school training institutions of compulsory education in Hefei have been closed, and only 57 are left with a survival rate of 4.75% (Yu and Yao, 2022). The Hefei Education Bureau issued the supporting rules of the “double reduction” policy in order to change the status quo of excessive pressure and burden on primary and secondary school students and their parents. The policy has been put in place, and is becoming more and more specific, and has achieved significant results in controlling the risk of primary and secondary school students (referred to as school students herein)’ suicide. However, the anxiety of students’ parents is obviously diversified and increases incrementally, such as “target panic” anxiety, “lack of means to make up for the shortcomings” anxiety, “lack of rescue path” anxiety when facing the decline of their children’s performance at school, and “bewildered” educational anxiety due to the lack of sensitivity of the transformation of the academic evaluation system. These gradually fill the hearts of parents to the depths of their minds, triggering a new type of “involution” in basic education. At the same time, parents’ anxiety can spread within family and even externally to a certain extent, and can negatively affect school students’ education through intergenerational transferability, feeding students’ misbehavior at school (Bögels et al., 2013; Conners-Burrow et al., 2015) and seriously affecting the physical and mental health of the majority of adolescents. It can be said that either the success or failure of the “load reduction” could lead to parents’ anxiety. Whether PAE is unrelated to the success or failure of “load reduction” goal and how to avoid repeating the outcome of the “double reduction” policy of South Korea in China have been becoming an urgent reasearch question for educational scholars.

The key to not repeating the historical trajectory of “double reduction” policy of Korea is to clarify the determining factors of PAE, explore the mechanism of interaction between the factors, and quantify PAE to provide theoretical basis and data support for PAE intervention and anxiety risk management policies. Therefore, the study of the MQPAE has become a top priority. This study can help solving the diversified anxiety problems of parents, meet the practical needs of “double reduction,” and has theoretical significance and practical value.

## Literature review

The following research progress is described in terms of both the concept of educational anxiety and the research on the measurement of educational anxiety.

Anxiety is a spiritual psychological concept that Freud viewed as a warning presented when the ego feels threatened. Jacobson developed Freud’s theory of anxiety as a signal indicating the approach of danger from the anxious self to its interior (Hautmann et al., 2015). Educational anxiety has received attention from many scholars as a collective manifestation of anxiety research in the field of education (May, 2010). In the 1970s, Sarason and Gardner proposed the problem of educational anxiety targeting different types of subject populations such as students (Mandler and Sarason, 1952; Sarason, 1978), teachers, and parents, and then it was developed rapidly. Before the “double reduction” policy in China, there were many cases of school students falling from buildings caused by educational anxiety, for which parents’ anxiety was more responsible, such as parents’ oppression (parents’ concern about their children’s educational results), bewilderment (parents’ concern about the educational process) and excessively ideal (depending mainly on family capital) education of their children. Parental anxiety has diversified after the “double reduction” policy, and it can be seen that parental anxiety has become the typical of educational anxiety.

Parental anxiety affects educational anxiety to a great extent, and may even spread and expand within family or even outside, causing negative effects on school students’ education and seriously affecting the physical and mental health of adolescents. Therefore, it is imperative to understand the emotional presentations of parental anxiety on their children’s education, empirically verify its mechanisms, explore the precise measurement of parental anxiety, and provide data support for dynamic management and implementation of parental anxiety intervention behaviors. However, anxiety measurement is still in its infancy in the academic community, and the self-assessment scale of anxiety (SAS) and state–trait anxiety inventory (STAI) were more often used to measure parental anxiety in the early stage (Kvaal et al., 2010; Sun et al., 2020). These instruments were deemed to ignore the boundary issues of educational measurement in terms of the stability of application with a certain degree of lack of academic rigor, and to obtain very rough results with low credibility. In 2018, Han Haitang examined PAE from five dimensions: school selection, school performance, learning attitude, parent–child interaction and attitude development and obtained the “PAE Scale” (Han, 2019). In the same year, Li Lin expressed PAE in three dimensions: employment anxiety, examination anxiety and health anxiety, and obtained the Educational Anxiety Questionnaire (Li, 2018); in 2019, Cheng Fangqi proposed a questionnaire on parents’ anxiety about their children’s education in three dimensions: achievement anxiety, own educational ability anxiety and health anxiety (Cheng, 2020). The above questionnaires or scales were constructed with differences in terms of measurement criteria, and their connotation and extension of the concepts were unclear and needed to be further clarified. In 2021, Li Jinzhou and Liu Yanmei compiled their own parental anxiety questionnaires and conducted studies related to the measurement of anxiety among parents of school students (Li, 2021; Liu, 2021), which also had differences in focus and lacked uniformity in the results obtained. By reviewing the domestic and international literature, it was found that research on the measurement of PAE is still not standardized, and application research on the measurement is relatively rare.

## Research hypothesis

Current research has shown that school performance is the core educational issue that parents care about their children (Zhou, 2021), and good or bad grades are directly related to parents’ psychological emotions (Li, 2021), and the degree of psychological health directly affects individual behavior. Based on the above findings, Hypothesis 1 is proposed.

*Hypothesis 1*: School performance affects parental mood and school performance anxiety is associated with parental anxiety, controlling for demographic sociological characteristics variables.

The results of existing studies show that students with good attitudes toward learning have relatively superior school performance (Han, 2019), and there is a direct correlation between the good school performance and the level of parental anxiety (Liu, 2021); therefore, we propose Hypothesis 2.

*Hypothesis 2*: School performance depends on students' attitudes towards learning and the attitude relates to parental anxiety levels.

Current research has found that the scarcity of quality teachers triggers educational anxiety, and the competition for school districts and quality schools still exists. At the same time, phenomenons such as “target-to-school work,” “students with special skills,” and “point recruitment” still exist (Yu and Yao, 2022), and competition has never disappeared, so hypothesis 3 is proposed.

*Hypothesis 3*: There is an association between parental anxiety and school choice for further education.

The results of existing studies reveal that parents’ educational competence is related to the degree of parent–child interaction (Han, 2019), which in turn is related to the degree of parents’ understanding of school students’ learning and affects parents’ knowledge of their children’s learning status. Therefore, Hypothesis 4 is proposed.

*Hypothesis 4*: Educational capacity and parental anxiety are related.

Both the competition for quality learning opportunities and the arrangement of the educational environment are inseparable from the involvement of family capital. No matter it is the purchase of houses in quality school district before the “double reduction” policy, or “High-level housekeeping,” “crowd funding private tutoring” and “study tours” after the policy, all of them are inseparable from family capital investment (Han, 2019). We propose hypothesis 5.

*Hypothesis 5*: There is a correlation between family capital and parental anxiety.

## Materials and methods

### Initial questionnaire development

PAE can be defined as an unpleasant emotional experience or emotional disorder formed by parents’ psychological feelings of inner tension, unease, anxiety, worry, concern and fear when facing the threat of learning environment, school performance and the pressure for higher education of their children. Risk sources of parental anxiety can be constructed with reference to risk perception theory covering three major sections: threat perception of competition in learning environment (school selection and employment competition), risk perception (learning attitude and school performance pressure) and thinking and application ability (educational capacity and family capital).

**Figure. 1:**
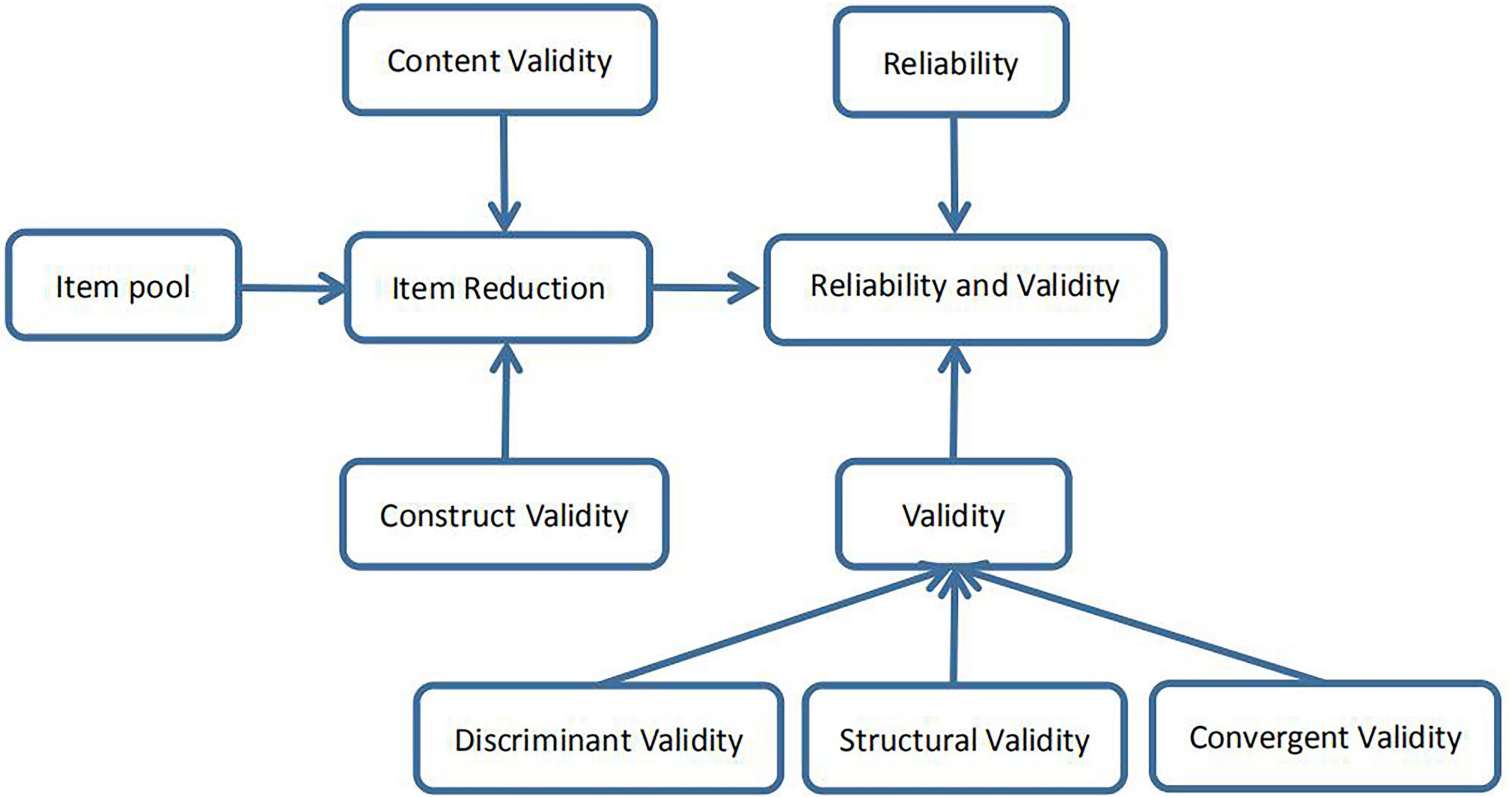
Research roadmap.

The overall purpose of the MQPAE was to clarify the level of PAE. In terms of threat perception, it is based on the competitive threat that may be brought to individuals by school selection and employment opportunities; in terms of risk perception, it is based on learning attitude and school performance to experience educational pressure and competitive risk; in terms of thinking and application ability, it is based on educational capacity and family capital to characterize parents’ educational thinking and application. According to the overall objective of the questionnaire, we selected some items from the existing relevant questionnaires through literature research. Combining with the experience of scholars in the field of constructing questionnaires and the results of expert interviews, a total of 46 initial measurement items were finally formed. The project team appropriately revised the above item factors through mechanistic analysis, separated the overlapping information between factors, and obtained a pool of 40 questionnaire items. In order to improve the item content validity and construct validity, we invited risk management experts, psychologists and health management scholars to brainstorm the items one by one to further clarify the structural relationships of the measurement indicators and to form the basic version of the MQPAE. It mainly includes 6 themes with 35 candidate items: (1) school performance (9 items); (2) attitude toward learning (6 items); (3) choice of school for further education (8 items); (4) employment (4 items); (5) educational capacity (5 items); and (6) family capital (3 items). On this basis, we conducted a pre-survey using online research with subjects ranging in age from 33 to 66 years old including 26 males and 62 females. Some of the items were deleted and integrated based on the pre-survey results, and the initial questionnaire on PAE was developed which consisted of 32 items covering six factors. According to the Likert five scale, there are five options for each item, i.e., “very incompatible, basically incompatible, unclear, basically compatible, and completely compatible,” and each item is rated on a scale of one to five. The specific research roadmap is shown in Figure 1 (Sun et al., 2022).

### Participants

A random sampling method was used to conduct the survey. There were 32 items in the initial questionnaire, and an appropriate sample size should be 10 to 20 times the number of items (Sun et al., 2018). Between June 15, 2022 and July 14, 2022, data on school students’ parents’ perceptions of educational anxiety were collected from primary and secondary schools in Anhui Province, and a total of 6,404 questionnaires were collected with 11 self-tested invalid questionnaires being excluded, and 6,393 valid questionnaires left, which results in a valid response rate of 99.83% meeting the sample size requirement. Among all respondents, 2,022 were males and 4,371 were females. The subjects’ average age was 42.71 (SD = 4.817), with a majority of 5,888 people aged 35–50 years.

### Data collection and quality control

A web-based survey was used to collect the data, and the teachers in charge of the surveyed schools were given uniform training on the specifications for completing the questionnaire before the survey. The survey was conducted by teachers who were employees in the target primary and secondary school and obtained informed consent from the study participants by means of parent-teacher conferences, and the link of the questionnaire was sent on the spot by the parent-teacher conference presenters. In case of doubt, the researcher was asked in person, and the questionnaire took approximately 8 min to complete, and subjects participated voluntarily. The collected questionnaires were logically verified, and those with obvious logical errors were eliminated. This study was approved by the Ethics Committee of Anhui Medical University.

### Statistical analysis method

We randomly divided the sample into two equal parts at a ratio of 1:9 with 5,754 questionnaires subjected to factor analysis and 639 to a reliability study. In this study, the discrete trend method, *t*-test, and Pearson’s correlation coefficient method were used for initial screening of PAE items. Standard deviations obtained by the discrete trend method were used to reflect the discrete trend of each item. The smaller the standard deviation the worse its discrimination, and the discrimination is generally considered bad and should be deleted when the standard deviation is less than 0.8 (Jiang, 2022). The critical ratio of *t*-test is the cut-off value between the acceptance and rejection domains. It is usually considered that reliability with a critical ratio greater than 3.0 for an item is acceptable, and the higher the critical ratio value, the better the differentiation of the item. Otherwise, items should be removed with a critical ratio less than 3.0 or no statistical difference (Chen, 2009). Pearson’s correlation coefficient method showed that a low correlation coefficient indicates a weak association which is usually judged by 0.4, and items were deleted with r less than 0.4 (Chen, 2009; Jiang, 2022). On this basis, exploratory factor analysis (EFA) was conducted to perform the final screening of the questionnaire items and reliability (Kim et al., 2016). In this study, the retention and rejection of items are mainly based on the following criteria: (i) if the maximum factor loading in the factor analysis is less than 0.4 (Chen, 2009; Jiang, 2022); (ii) if an item spans two or more factors and the difference between the maximum factor loading and the second largest factor loading of the item is less than 0.2, the item is rejected (Chen, 2009; Jiang, 2022); (iii) if the number of items contained under the common factor of an item is less than three, the factor and the items contained in it are rejected (Chen, 2009; Jiang, 2022). The reliability of the questionnaire was assessed by internal consistency and Pearson’s correlation coefficient analysis. The Cronbach’s α coefficient method was used to test the internal consistency reliability of the questionnaire (Thom et al., 2011). It is generally believed that the closer the coefficient is to one, the better the internal consistency of the questionnaire and the higher the homogeneous reliability. The reliability of the retest was examined by conducting a second survey using the final version of the questionnaire after a certain period of the first survey. The Pearson’s correlation coefficients (retest reliability) for the total questionnaire and for both pre and post-scores of each factor were calculated and the values of the parameter r indicated the retest reliability of the total questionnaire. A correlation coefficient which was greater than 0.7 and statistically significant is considered good (Gliner et al., 2001; Jones et al., 2015). Confirmatory factor analysis (CFA) was conducted using 639 pre-selected data to investigate the validity of the questionnaire. Structural equation modeling was used to investigate the structural validity of the questionnaire, and the fit indicators were χ^2^/df, RMSEA, GFI, NFI, RFI, and CFI. For example, when χ^2^/df was less than five, the structural model was acceptable and the closer χ^2^/df was to zero, the better the overall fit of the model. Discriminant validity and convergent validity were assessed by average variance extracted (AVE), combined reliability (CR), and maximum of shared squared variance (MSV) (Wang et al., 2010; Kim et al., 2015), and the factors had good convergent validity when AVE was greater than 0.5 and CR was greater than 0.7, and had good discriminant validity when MSV was less than the corresponding AVE and the square root of the factor AVE was greater than the correlation coefficient between the factor and other factors.

## Results

### Item screening and factor analysis

In this study, the discrete trend method was used to screen the questionnaire items. The results of data analysis are shown in Table 1.

**Table 1:** Results of the discrete trend method analysis.

Items	Mean	*SD*	Items	Mean	*SD*	Items	Mean	*SD*
Item 1	3.32	0.909	Item 12	3.45	1.020	Item 23	3.22	1.115
Item 2	3.35	0.889	Item 13	3.57	1.013	Item 24	3.41	1.067
Item 3	3.71	0.926	Item 14	3.82	1.061	Item 25	3.17	1.034
Item 4	3.20	0.965	Item 15	3.55	1.066	Item 26	3.17	1.089
Item 5	3.35	0.986	Item 16	3.66	0.991	Item 27	3.18	1.062
Item 6	3.25	0.933	Item 17	2.95	1.064	Item 28	3.41	1.049
Item 7	2.84	1.048	Item 18	3.73	0.964	Item 29	3.07	1.064
Item 8	3.35	1.080	Item 19	3.41	1.002	Item 30	3.19	1.022
Item 9	3.02	1.044	Item 20	3.39	1.002	Item 31	3.52	0.972
Item 10	3.47	1.006	Item 21	3.61	1.033	Item 32	2.87	1.098
Item 11	3.62	1.051	Item 22	3.36	1.108			

Based on the above results, the standard deviation of the 32 items was greater than 0.8, the criteria for deleting items under the discrete trend method, so that no items was deleted.

We further analyze the critical value to check the credibility of the items. Subjects were ranked from the highest to the lowest based on total scores on educational anxiety, with those scoring in the top 27% as the high group and those in the bottom 27% as the low group. The mean of the scores of the high and low groups on each item was found, and the test of variance between the means of the two groups was conducted to remove items with a critical ratio less than 3.0 or no statistical difference (*p* > 0.05). The results of the analysis are shown in Table 2.

**Table 2:** Results of the *t*-test analysis.

Items	*t*	*p*	Items	*t*	*p*	Items	*t*	*p*
Item 1	−44.656	<0.001	Item 12	−63.660	<0.001	Item 23	−62.544	<0.001
Item 2	−46.083	<0.001	Item 13	−62.560	<0.001	Item 24	−65.304	<0.001
Item 3	−49.334	<0.001	Item 14	−52.933	<0.001	Item 25	−63.254	<0.001
Item 4	−35.530	<0.001	Item 15	−61.888	<0.001	Item 26	−60.822	<0.001
Item 5	−49.386	<0.001	Item 16	−66.636	<0.001	Item 27	−59.376	<0.001
Item 6	−51.629	<0.001	Item 17	−38.906	<0.001	Item 28	−68.984	<0.001
Item 7	−43.245	<0.001	Item 18	−63.916	<0.001	Item 29	−52.138	<0.001
Item 8	−63.399	<0.001	Item 19	−49.448	<0.001	Item 30	−45.451	<0.001
Item 9	−38.007	<0.001	Item 20	−61.871	<0.001	Item 31	−43.344	<0.001
Item 10	−66.863	<0.001	Item 21	−68.035	<0.001	Item 32	−23.252	<0.001
Item 11	−62.552	<0.001	Item 22	−61.395	<0.001			

The results of the decisive value analysis found the above items generally credible with no items being deleted.

We further used the Pearson’s correlation coefficient method to analyze the correlation between each item and the total score of the initial questionnaire. The obtained results are shown in Table 3.

It was found that Item 32 did not meet the requirements of the project (*r* = 0.366 <0.4), therefore, this item was deleted.
We performed KMO and Bartlett’s test on the initial questionnaire for 31 items. The KMO value was 0.963 and the Bartlett’s spherical test chi-square value reached a significant level (*p* < 0.001), indicating that the data are suitable for EFA. We further analyzed the data to obtain the rotated component matrix is shown in Table 4, and five common factors with a root greater than one are extracted.

**Table 3:** Results of the Pearson’s correlation coefficient analysis.

Items	*r*	Items	*r*	Items	*r*
Item 1	0.610**	Item 12	0.748**	Item 23	0.735**
Item 2	0.632**	Item 13	0.747**	Item 24	0.759**
Item 3	0.641**	Item 14	0.656**	Item 25	0.739**
Item 4	0.516**	Item 15	0.731**	Item 26	0.723**
Item 5	0.661**	Item 16	0.775**	Item 27	0.719**
Item 6	0.692**	Item 17	0.554**	Item 28	0.775**
Item 7	0.610**	Item 18	0.758**	Item 29	0.678**
Item 8	0.742**	Item 19	0.655**	Item 30	0.609**
Item 9	0.545**	Item 20	0.740**	Item 31	0.588**
Item 10	0.773**	Item 21	0.774**	Item 32	0.366**
Item 11	0.736**	Item 22	0.731**		

^**^, *P* < 0.01.

**Table 4:** Component matrix after rotation.

Items	Components				
	1	2	3	4	5
Item 1		0.619			
Item 2		0.713			
Item 3		0.623			
Item 4		0.655			
Item 5		0.679			
Item 6		0.667			
Item 7		0.476			
Item 8	0.533	0.458			
Item 9					
Item 10	0.601	0.474			
Item 11	0.758				
Item 12	0.701				
Item 13	0.713				
Item 14	0.660				
Item 15	0.712				
Item 16	0.578				
Item 17					0.612
Item 18	0.494			0.502	
Item 19				0.494	0.544
Item 20				0.543	0.455
Item 21				0.711	
Item 22				0.738	
Item 23				0.678	
Item 24				0.676	
Item 25			0.654		
Item 26			0.681		
Item 27			0.754		
Item 28			0.628		
Item 29			0.692		
Item 30					0.733
Item 31					0.740

From Table 4, among the 31 items, the factor loading of Item 9 was less than 0.4, while the factor loading of the remaining items was above 0.4, therefore Item 9 was deleted. Item 8, Item 10, Item 18, Item 19, and Item 20 all spanned 2 factors and the difference between the two major factor loading was less than 0.2, indicating that these two items were significantly expressed on both factors with low specificity, so that these five items were deleted.

### Reliability analysis

EFA extracted five factors with 25 items, and the cumulative variance contribution was 65.66%. The total Cronbach’s α coefficient of the questionnaire was 0.956, and each factor was noted as F1, F2, F3, F4, and F5, and their Cronbach’s α coefficients were 0.926, 0.857, 0.913, 0.901, and 0.768, respectively. The collection of data for retest reliability was conducted 45 days after the initial survey, using the final version of the questionnaire with a secondary survey of 722 parents randomly selected from those initially surveyed (August 28, 2022 to September 7, 2022). 534 valid data were extracted based on IP matching of mobile phone numbers, and the scores of both pre-and post-surveys were calculated. The Pearson’s correlation coefficients of the total questionnaire and of the five factors were 0.908, 0.911, 0.873, 0.891, 0.907, and 0.885, respectively (all *p* < 0.001). Higher scores for the total questionnaire indicate higher levels of anxiety.

### Validity analysis

In the following, the reliability of PAE measures in terms of structural and discriminant validity was empirically validated.

### Construct validity

**Figure 2:**
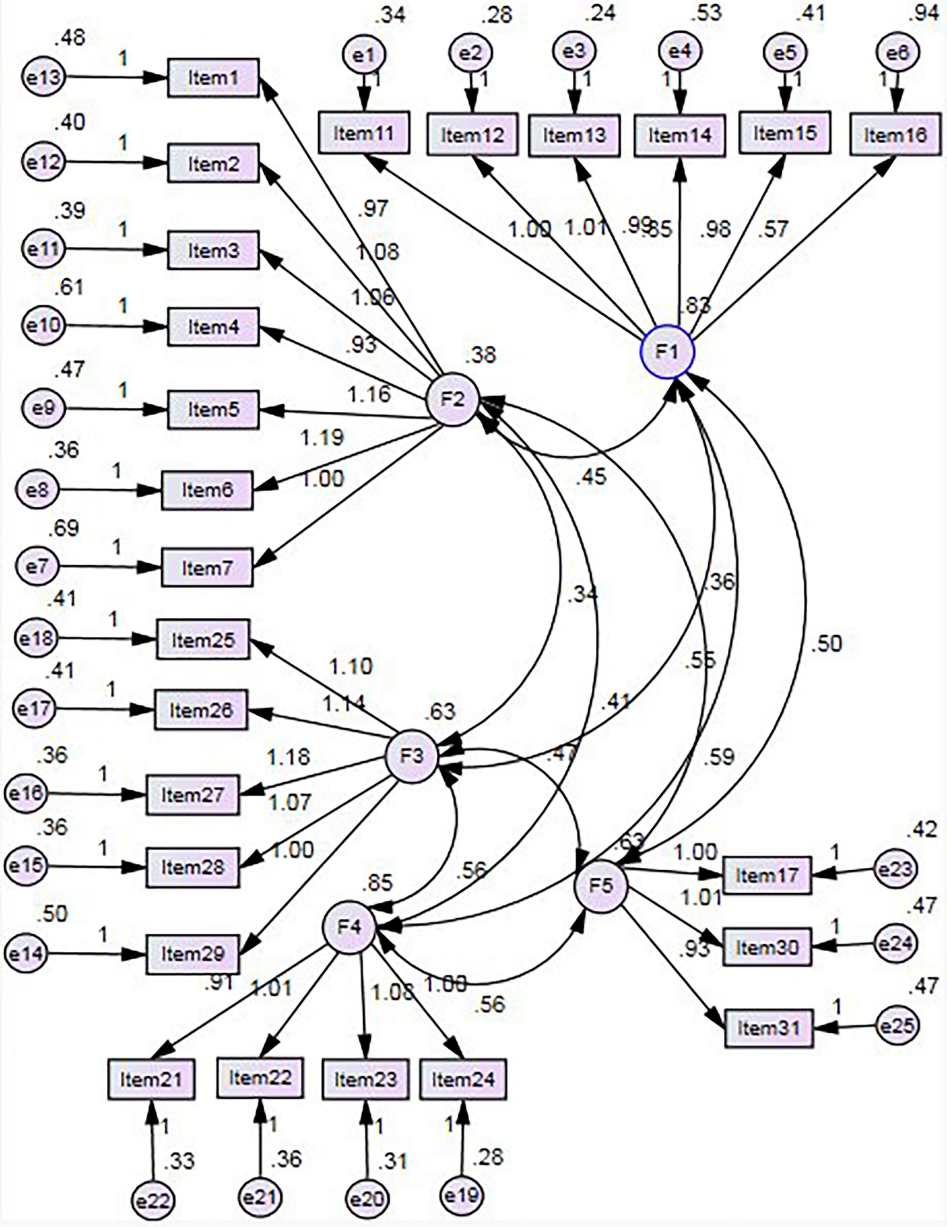
Structural equation model for the MQPAE.

In this study, CFA was used to test whether the factor model of the formulated questionnaire was consistent with the theoretical framework constructed in the previous study. The results obtained were more scientific and reasonable by testing the theoretical model with empirical data. In this study, the validation analysis was conducted using 639 pre-selected data, and the above-mentioned evaluation indicators of the MQPAE were: χ^2^/df = 4.306, AGFI = 0.839, GFI = 0.869, TLI = 0.909, CFI = 0.920, IFI = 0.920, NFI = 0.898, and PGFI = 0.708, PNFI = 0.793, and RMSEA = 0.072. According to the discriminant criteria in the literature, it shows that the structural model of the questionnaire in this study has a good fit. The structural equation model plot is shown in Figure 2.

### Discriminant validity

The standard loading coefficient for each item was obtained by CFA (Table 5).

**Table 5:** Coefficients for factor loading.

Factors	Items	Non-standard loading factors (Coef.)	Standard error (Std. Error)	*Z*	*p*	standard loading factors (Std. Estimate)
F1	Item11	1.000				0.842
	Item12	1.012	0.036	27.889	0.000	0.868
	Item13	0.989	0.035	28.538	0.000	0.880
	Item14	0.849	0.040	21.190	0.000	0.727
	Item15	0.978	0.039	24.993	0.000	0.812
	Item16	0.568	0.046	12.301	0.000	0.741
F2	Item7	1.000				0.598
	Item6	1.187	0.079	15.034	0.000	0.774
	Item5	1.157	0.086	14.378	0.000	0.724
	Item4	0.930	0.075	12.455	0.000	0.594
	Item3	1.058	0.074	14.376	0.000	0.724
	Item2	1.084	0.075	14.423	0.000	0.727
	Item1	0.967	0.072	13.361	0.000	0.652
F3	Item29	1.000				0.745
	Item28	1.066	0.051	20.918	0.000	0.816
	Item27	1.176	0.054	21.635	0.000	0.841
	Item26	1.142	0.055	20.898	0.000	0.815
	Item25	1.105	0.054	20.641	0.000	0.806
F4	Item24	1.000				0.869
	Item23	1.078	0.037	29.329	0.000	0.871
	Item22	1.014	0.037	27.508	0.000	0.840
	Item21	0.914	0.034	26.769	0.000	0.827
F5	Item17	1.000				0.775
	Item30	1.013	0.054	18.608	0.000	0.752
	Item31	0.933	0.052	17.962	0.000	0.735

Table 5 showed that the standard loading coefficients were greater than or close to 0.6, therefore the relationship between the items and their respective factors was reasonable and met the criteria for item selection.

The results of the CFA were presented in Table 6.

**Table 6:** Pearson’s correlation and AVE square root values.

	F1	F2	F3	F4	F5
F1	0.976				
F2	0.799***	0.937			
F3	0.755***	0.691***	0.969		
F4	0.704***	0.712***	0.767***	0.982	
F5	0.695***	0.726***	0.741***	0.765***	0.970

The shaded figures are the square root values of the AVE. ***p < 0.001.

Table 6 shows that the factor F1, F2, F3, F4, and F5 are significantly correlated with each other (*p* < 0.001) and all of the correlation coefficients are less than the square root value of the corresponding AVE, which means that the latent variables are correlated with each other and are distinguished from each other.

To further analyze the convergent and discriminant validity of the questionnaire, we obtained information on the CR and MSV, see Table 7.

**Table 7:** CR and MSV.

	AVE	CR	MSV
F1	0.953	1.000	0.453
F2	0.878	1.000	0.442
F3	0.938	1.000	0.450
F4	0.964	1.000	0.472
F5	0.942	1.000	0.451

AVE, average variance extracted; CR, combined reliability; MSV, maximum of shared squared variance.

Table 7 displays that the AVE was greater than 0.5 and the CR was greater than 0.7, therefore the questionnaire was of good convergent validity, and MSV was less than the corresponding AVE which further indicated that the questionnaire had good discriminant validity.

## Discussion

The results of the discrete trend method and the decisive value analysis method show that the initial items of the MQPAE are reasonable. One of the possible reasons for this comes from the reasonableness of our process of constructing the item pool. Coupled with the pre-survey analysis, the constructing process eliminated some of the unreasonable items. The second reason stems from the fact that these two methods are suitable for roughly eliminating abnormal items.

The results of Pearson’s correlation study show that the correlations between the 32 items and their total scores were statistically significant, and the correlation coefficients were greater than 0.5 except for Item 32. Since the Pearson’s correlation coefficient method is higher in terms of the precision of items than the discrete trend method and the decisive value analysis method, it is beneficial to further optimize the questionnaire item pool. The results of EFA show that Item 8, Item 9, Item 10, Item 18, Item 19, and Item 20 did not meet the requirements of the questionnaire factor composition, which optimized the initial questionnaire from a statistical perspective and improved the quality of the MQPAE.

Reliability is expressed by the Cronbach’s α coefficient, which measures the homogeneity or intrinsic correlation between the items and evaluates the accuracy, consistency and stability of the questionnaire as well as the degree of variability in the measured values due to random errors during the measurement process (Thom et al., 2011). In general, a Cronbach’s α coefficient higher than 0.8 for the total questionnaire and higher than 0.6 for each dimension indicates that the questionnaire has good reliability (Thom et al., 2011). The overall Cronbach’s α coefficient of the MQPAE was 0.956 higher than 0.8, and the Cronbach’s α coefficients for the five factors were 0.926, 0.857, 0.913, 0.901, and 0.768, respectively, all of which were greater than 0.7, indicating that the scale reliability was good and the internal consistency of each factor was acceptable, implying that the questionnaire had good internal consistency (Chen, 2009; Jiang, 2022). The results of the retest show that the Pearson’s correlation coefficients r for the total questionnaire and for the five factors were 0.908, 0.911, 0.873, 0.891, 0.907, and 0.885 respectively, further indicating that the reliability of the retest of MQPAE was good. This is related to the rigor and scientificity of our research process.

Five common factors (with eigenvalues greater than one) were extracted by principal component analysis, with a cumulative variance contribution of 65.66%. In the CFA, the ratio of χ^2^ value to the degree of freedom *df* is usually used to indicate the probability of correctness of the structural model, and the smaller the ratio, the better the model fit. When χ^2^/*df* is less than five, it indicates that the structural model is acceptable, and the closer χ^2^/*df* is to zero, the better the overall fit of the model (Wang et al., 2010). For this questionnaire, χ^2^/*df* is equal to 4.306 less than five, which indicates that the questionnaire structural model meets the criteria. In addition, Comparative fit index (CFI), normed fit index (NFI), adjusted goodness of fit index (AGFI), Root mean square error of approximation (RMSEA) error of approximation (RMSEA), Parsimony normed fit index (PNFI), and Parsimony goodness of fit index (PGFI) are also often used to measure the fit of factor models. CFI, NFI, and RMSEA vary between 0 to 1. As for CFI, NFI, being closer to one indicates a better fit (Chen, 2009; Jiang, 2022). AGFI is greater than 0.80 indicating a better model fit (Chen, 2009; Jiang, 2022). RMSEA should be less than 0.08, and the smaller the value, the better the fit (Chen, 2009; Jiang, 2022). PNFI and PGFI are greater than 0.50, implying a good model fit (Chen, 2009; Jiang, 2022). In this study, CFI = 0.920, NFI = 0.898, RMSEA = 0.072<0.08, AGFI = 0.893>0.80, PNFI = 0.793, and PGFI = 0.708, all of which met the model fit requirements, indicating a good fit of the questionnaire structure model. CFA yielded standardized loading coefficients greater than or close to 0.6 for each item, which is sufficient to show that the relationship between the items and their respective factor was reasonable and met the criteria for item selection (Chen, 2009), which is related to the completeness of our study process.

The square root of the AVE indicates the “convergent” nature of the factor, and was compared with the correlation coefficients of other factors to investigate the discriminant validity of the MQPAE. The correlation coefficient indicates the correlation relationship, and if the factor is highly “aggregated,” i.e., the AVE square root value is greater than the “correlation coefficient between the factor and other ones,” and MSV was less than the corresponding AVE, then it indicates the discriminant validity (Wang et al., 2010; Kim et al., 2015; Chi and Heesup, 2020). The AVE was greater than 0.5 and the CR was greater than 0.7, which presents the MQPAE has good convergent validity. The combined results of the series reveal that the MQPAE has good convergent validity and discriminant validity.

The questionnaire factors and item content presented in this study as follows (see Table 8).

**Table 8:** Factors and items of the questionnaire.

Factors	Items
F1	Item 11,You are worried about children’s lack of learning initiative
	Item 12, You are anxious about children’s fear of difficulty
	Item 13, You are worried about children’s lack of interest in learning
	Item 14, You are worried about children’s neglect of learning due to playing with cell phones
	Item 15, You are worried about children’s low efficiency in completing homework
	Item 16, You are worried about children’s failure to get into good schools
F2	Item 1, You feel anxious about your child’s school performance
	Item 2, You feel nervous when checking your child’s performance
	Item 3, You worry that your child’s school performance will drop
	Item 4, You compare your child’s performance with that of other students in the same class
	Item 5, You get anxious about your child’s learning competition when you see other children taking extra classes
	Item 6, You feel nervous when your child is about to take an exam
	Item 7, You often give instructions to your child on homework and worry or panic because you know your child’s school performance
F3	Item 25, You feel annoyed because your child does not like to talk to you about learning
	Item 26, You feel annoyed because you often have conflicts with your child about learning
	Item 27, You feel annoyed because you do not know how to communicate and get along with your child
	Item 28, You feel annoyed because you do not know how to help your child learning
	Item 29, You feel annoyed because you do not have time to communicate with your child about learning
F4	Item 21, You worry that the child will not be able to find a satisfactory job
	Item 22, You worry that the child’s future financial income will not be able to support a large amount of expenses such as purchasing a house or a car
	Item 23, You worry that the child’s future economic income and social status will be lower than their current level
	Item 24, You worry that the child will not be able to adapt to the future competitive social environment
F5	Item 17, You would consider paying a large amount of fees for school choice if it is difficult for your child to receive further education
	Item 30, You would do everything possible to bring your child into the desired class
	Item 31, You would do everything possible to give your child access to quality teachers

Item 11, Item 12, Item 13, Item 14, and Item 15 are directly related to learning attitudes. Item 16 reflects the issue of choosing schools for further education. Although Item 16 is not directly related to learning attitudes, children’s learning attitudes determine learning performance (Liu, 2021), and performance determines the initiative of choosing schools for further education, therefore Item 16 is indirectly related to learning attitudes. In other words, PAE was related to their children’s learning attitudes and indirectly to their opportunities of choosing desired schools for further education. This verifies hypotheses 2 and 3. F2 contains 7 items, which directly respond to the child’s school performance, and the results of the study show that information on the child’s school performance items was significantly associated with PAE. This is in line with the findings of the literature (Li, 2021). Li pointed out that good or bad grades are directly related to parents’ psychological emotions. This confirms hypothesis 1. F3 contains five items, which directly reflect parental educational competence. The correlation coefficients between the total scores of the MQPAE and the items reflecting parents’ educational capacity were within the range of 0.678 to 0.775 (all *p* < 0.001), and the findings show that parental educational competence is significantly associated with PAE, confirming Hypothesis 4. F4 contains 4 items, which can directly reflect the child’s economic income level, and are also considered some concrete manifestations of the educational outcomes. The previous study pointed out that the level of education determines the future income level (Yu and Yao, 2022), and it is directly related to school choice, therefore it further confirms hypothesis 3. F5 contains 3 items, which are measures for these items are no more than buying a house in school district, going through the green channel for students with special skills, etc., and these routes are costly and require a certain amount of household capital, which is consistent with the findings of other literature (Han, 2019). Han pointed out that the investment of household capital in children’s education allows access to advantageous educational opportunities, thus confirming hypothesis 5.

## Conclusion

Through our research, the MQPAE, a tool for measuring anxiety of parents of primary and secondary school students, was proposed containing 5 factors and 25 items. Based on the connotation and significance of the items contained in each factor, we named F1 “learning attitude,” F2 “school performance,” F3 “educational capacity,” F4 “educational outcome,” and F5 “family capital.” The total Cronbach’s α coefficient of the questionnaire was 0.956, and the Cronbach’s α coefficients of each factor were 0.926, 0.857, 0.913, 0.901, and 0.768, respectively. The higher the overall score of the questionnaire, the higher the level of anxiety, the more likely it is that the person will develop an educational anxiety condition.

The MQPAE proposed in this study has good reliability and validity, and the application of this questionnaire can quantify the educational anxiety level of parents of primary and secondary school students. The implementation of this questionnaire can determine the influencing factors of parental anxiety, provide data guarantee and technical support for schools and educational administrations to carry out psychological interventions in order to solve the diversified anxiety problems of parents, improve the quality of home-school cooperation and meet the practical needs of the “double reduction” policy.

The generality of the MQPAE may be limited. Because of the huge potential differences in national conditions, for example, in terms of cultural and educational policies between different countries, the MQPAE may only be applicable to China’s scenarios.

## Data availability statement

The original contributions presented in the study are included in the article/supplementary material, further inquiries can be directed to the corresponding author.

## Ethics statement

This approval procedure was approved by the Ethics Committee of Anhui Medical University. Written informed consent for participation was not required for this study in accordance with the national legislation and the institutional requirements.

## Author contributions

JS designed this study and wrote the manuscript. JS, TL, YG, HL, YC, HD, GZ, HS, RC, and ZY collected data. TL and JS analyzed the data. JS, LL, and LZ provided guidance for statistical analysis, provided financial support, and reviewed the manuscript. All authors contributed to the article and approved the submitted version.

## Funding

This work was supported in part by the Hefei’s Provincial and Municipal Leaders Designated Research Topics in 2022 (SQKT202204), NSF Center for Basic Science Project (no. 72188101), Natural Science Foundation of Anhui Province of China (no. 1908085MG233), Quality Engineering for Research Projects of the Anhui Department of Education (nos. 2020SJJXSFK1341 and 2020wyxm108), Natural Science Foundation for the Higher Education Institutions of Anhui Province of China (nos. KJ2021A1228 and KJ2021A0266), and Projects for National Health Insurance Agency of the China (no. 2021056). Anhui Medical University School of Health Management “Advanced Mathematics for Medical Purposes” Course Civics Project.

## Conflict of interest

The authors declare that the research was conducted in the absence of any commercial or financial relationships that could be construed as a potential conflict of interest.

## Publisher’s note

All claims expressed in this article are solely those of the authors and do not necessarily represent those of their affiliated organizations, or those of the publisher, the editors and the reviewers. Any product that may be evaluated in this article, or claim that may be made by its manufacturer, is not guaranteed or endorsed by the publisher.
